# Chronic kidney disease on the background of bardet–biedl syndrome: a case report and review of literature

**DOI:** 10.1097/MS9.0000000000001626

**Published:** 2023-12-15

**Authors:** Pashupati Pokharel, Uday Pandey, Samir Sedai, Kapil Khanal, Midhan Shrestha

**Affiliations:** a Maharajgunj Medical Campus, Institute of Medicine, Tribhuvan University; b Department of Nephrology and Kidney Transplant Medicine, Tribhuvan University Teaching Hospital, Kathmandu Nepal

**Keywords:** bardet–biedl syndrome, case report, chronic kidney disease, genetic disorder, renal impairment

## Abstract

**Introduction:**

Bardet–Biedl syndrome (BBS) is a rare autosomal recessive multisystem disorder characterized by retinal dystrophy, obesity, postaxial polydactyly, renal dysfunction, learning difficulties, and hypogonadism. In this case report, the authors present the clinical course and management of a patient with BBS who developed chronic kidney disease (CKD).

**Case presentation:**

An 18-year-old male presented to the emergency department with chief complaints of fever, cough, vomiting, and decreased urine output for 7 days. Parents complained that the child had a delay in development compared to other children of the same age group. On examination, the patient had tachypnea, periorbital and pedal edema, expiratory wheeze with bilateral basal crackles, polydactyly, central obesity, microtestes, and delayed developmental milestones. Ultrasonography revealed bilateral small kidneys with increased cortical echotexture and loss of corticomedullary differentiation. Based on clinical features, the patient was diagnosed with CKD in the background of BBS. Hemodialysis was initiated after the diagnosis.

**Discussion:**

The management of CKD in the background of BBS poses unique challenges due to the complex multisystem involvement of this genetic disorder. There should be early reorganization and management of this condition so that the patient can have a better quality of life. Moreover, in developing countries like Nepal, genetic testing and diagnosis should be made easily accessible for better patient outcome.

**Conclusion:**

Multidisciplinary approach involving nephrologists, ophthalmologists, endocrinologists, and geneticists is important to optimize the treatment and long-term management of Badet Biedel patients.

## Introduction

HIGHLIGHTS

- Bardet–Biedl syndrome (BBS) is a rare autosomal recessive multisystem disorder.
- We report a case of BBS presenting with features of acute on chronic kidney disease in the emergency and managed with hemodialysis.
- Multidisciplinary approach is important to optimize the treatment and long-term management of the patient.

Bardet–Biedl syndrome (BBS) is a rare autosomal recessive multisystem disorder characterized by retinal dystrophy, obesity, postaxial polydactyly, renal dysfunction, learning difficulties, and hypogonadism^1^. It is caused by biallelic loss of function in at least 26 genes^2^. Current treatments for BBS are symptomatic, concentrating on aggressive management of diabetes, hypertension, and metabolic syndrome to reduce the secondary effects that these conditions have on vulnerable organ systems, particularly the eyes and kidneys^3^.

Chronic kidney disease (CKD) is defined as decreased kidney function shown by a glomerular filtration rate (GFR) of less than 60 ml/min per 1.73 m^2^, or markers of kidney damage, or both, of at least 3 months duration, regardless of the underlying cause^4^. According to a study conducted in United Kingdom (UK), the prevalence of CKD in patients with BBS is 31% in children and 42% in adults^5^.

Only a handful cases of BBS have been reported in Nepal. In this rare case report, we present the clinical course and management of a patient with BBS who developed acute CKD.

## Case presentation

**Figure 1:**
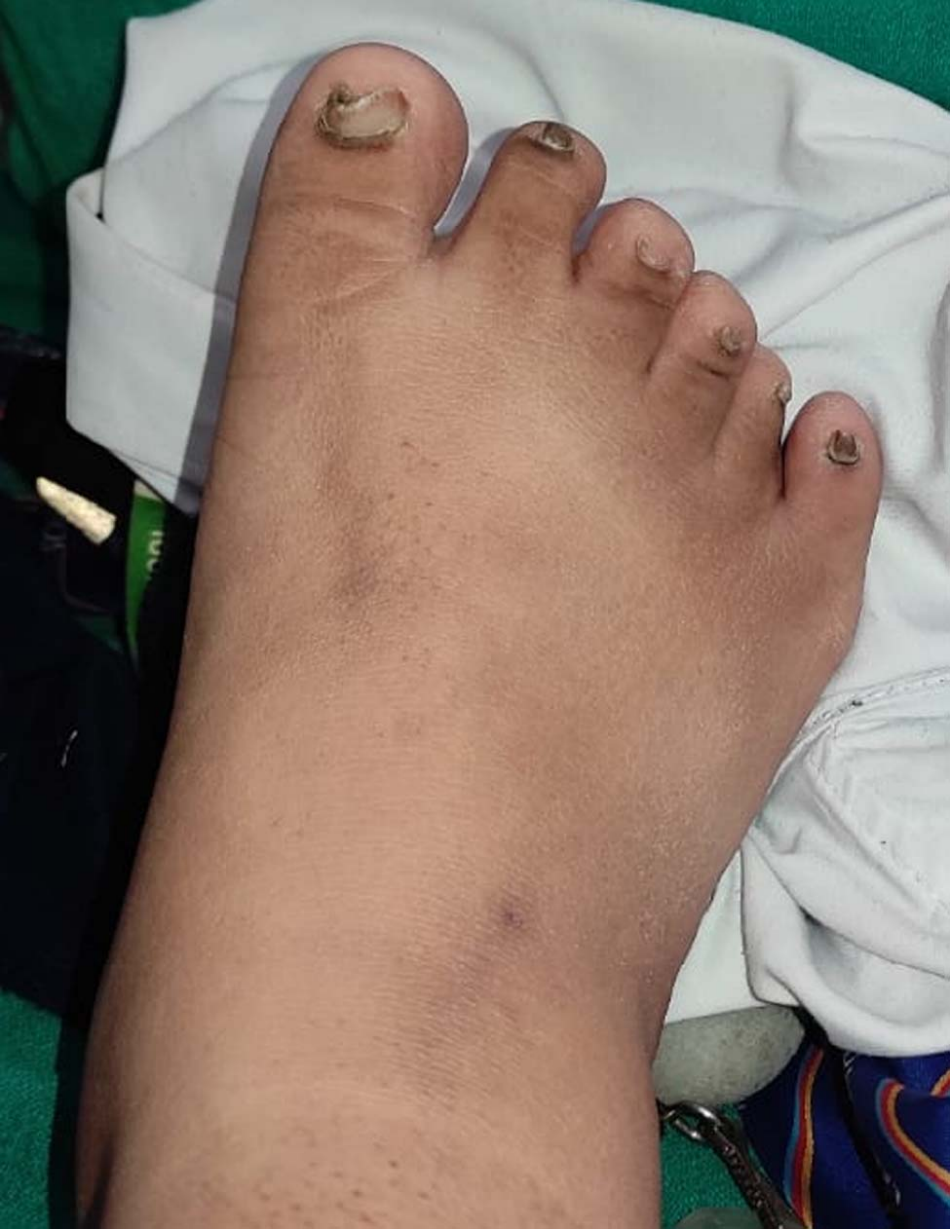
Polydactyly.

**Figure 2:**
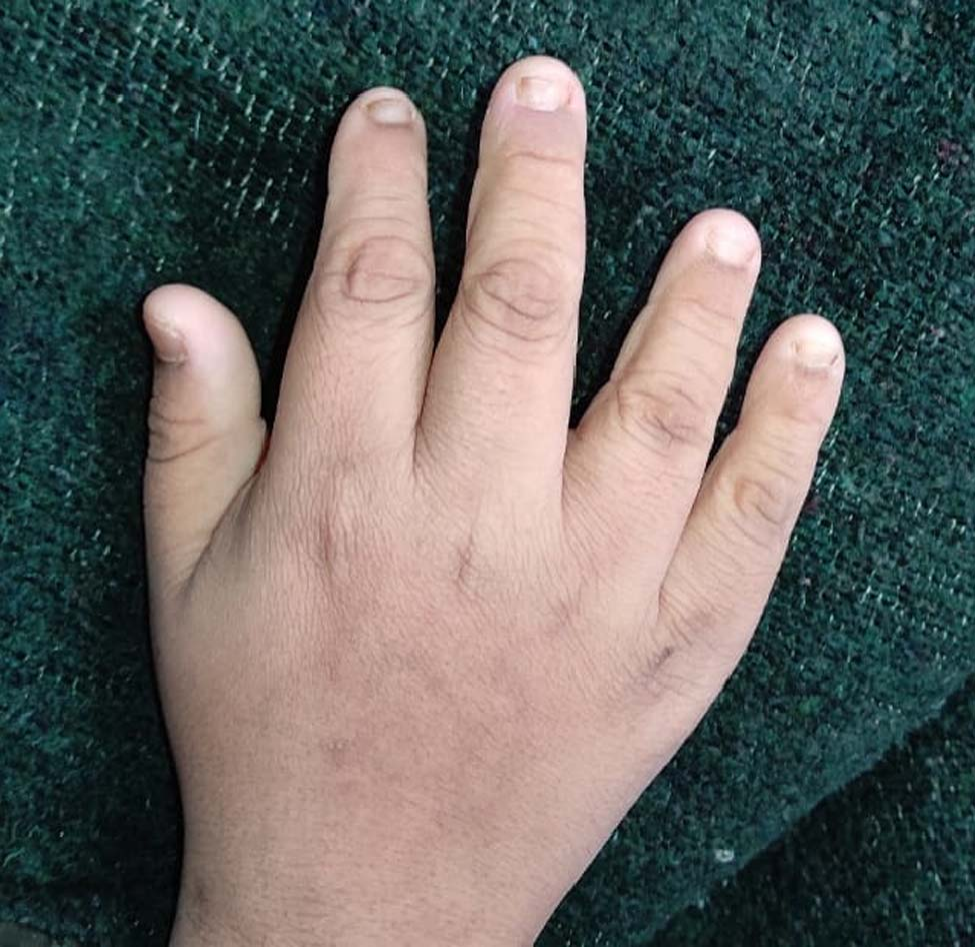
Brachydactyly.

**Figure 3:**
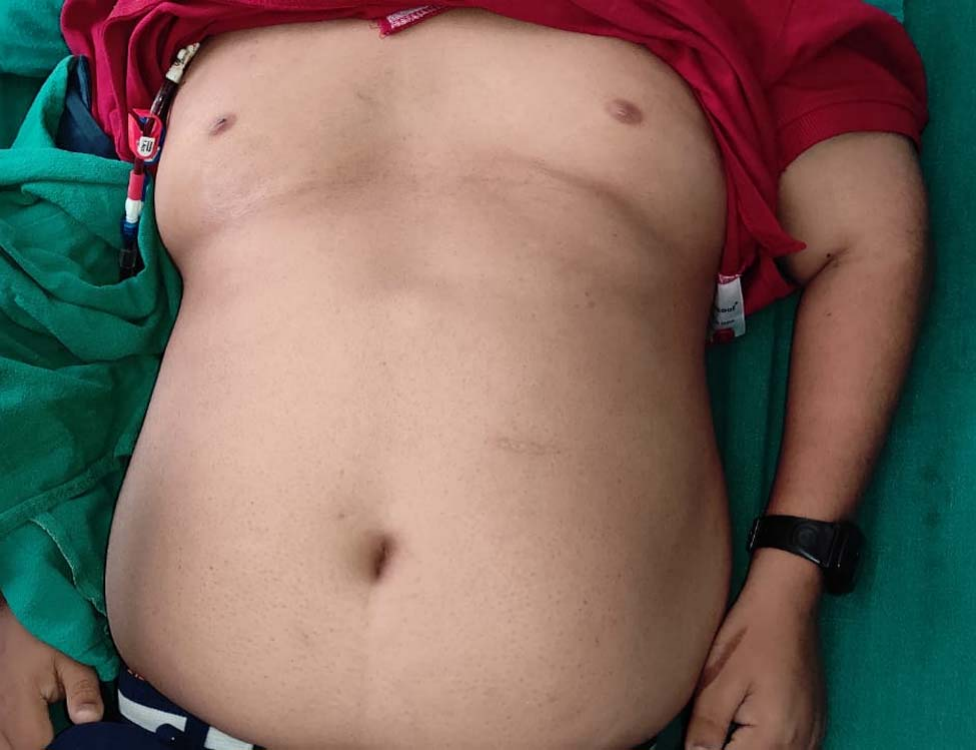
Abdominal obesity.

**Figure 4:**
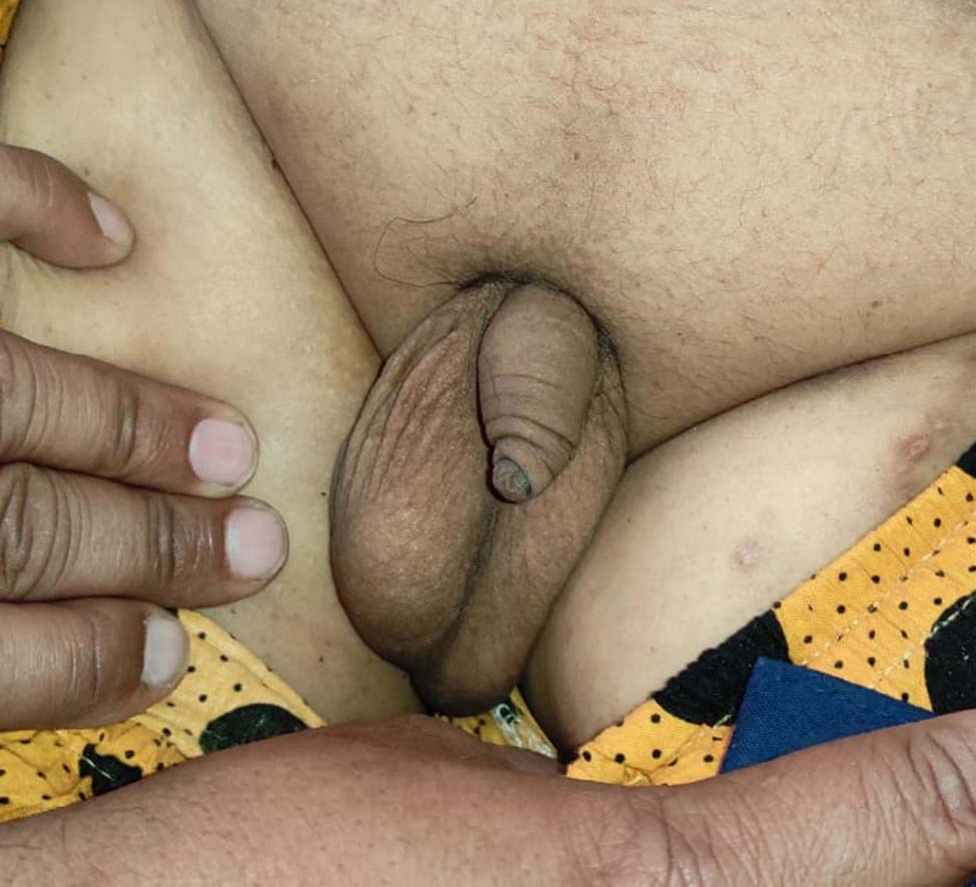
Microtestes with microphallus.

Our patient is 18-year-old male, the first offspring of nonconsanguineous parents. He was of normal weight when he was born but developed obesity in early childhood. The parents noticed delayed motor developmental milestones and learning disabilities. Digital abnormalities were also present since birth, these including polydactyly and brachydactyly. Examination revealed postaxial polydactyly in the bilateral lower limbs, and brachydactyly in the bilateral upper and lower limbs (Figs 1 and 2). Left upper limb also had an extra digit that fell off at the age of 12 years. He also had abdominal obesity (Fig. 3). The parents also noticed that his gonads had stopped developing since he was 6 months of age, and examination revealed hypogonadism in the form of microtestes and microphallus (Fig. 4). The patient had diminished nocturnal vision since birth, which progressed to complete blindness by the time he was 4 years old. The patient also had a history of recurrent childhood chest infections. His family history was also remarkable, with central obesity and decreased vision in his 14-year-old sister.

**Figure 5:**
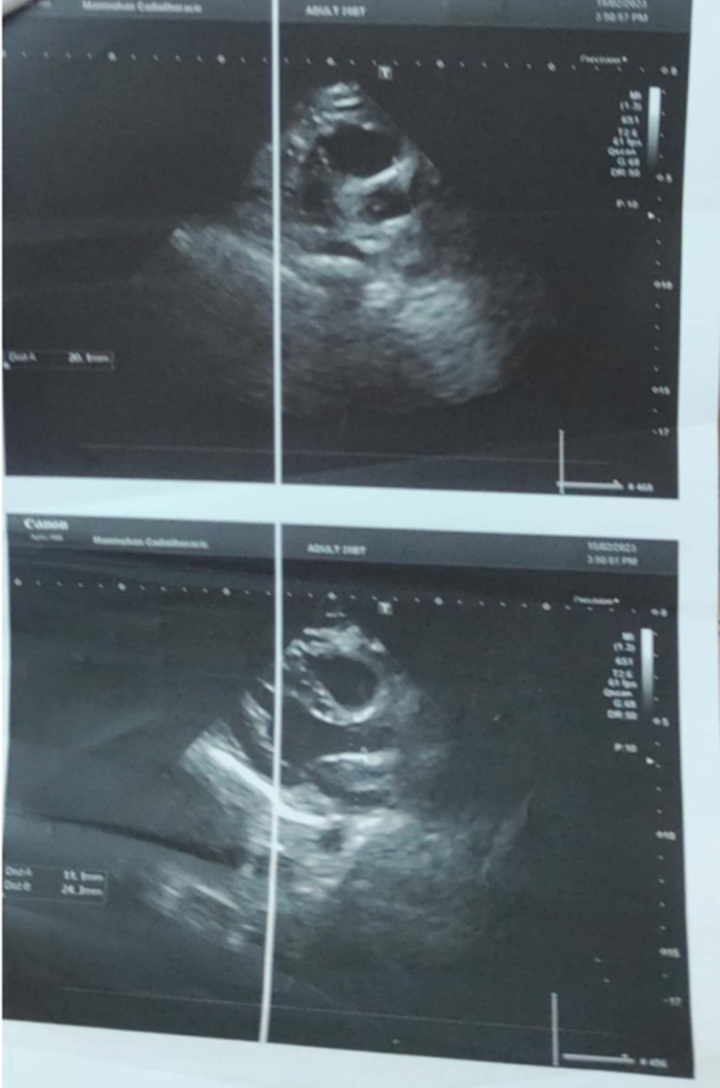
Ultrasonography showing features of chronic kidney disease.

The patient also gives history of polyuria, and nocturia for last 10 years. Additionally, he gives history of on and off type of frothiness in urine. Renal involvement was diagnosed when the patient was 16 years of age, with elevated urea and creatinine values (urea=70 mg/dl, creatinine=3.6 mg/dl). Ultrasonography showed bilateral small kidneys with increased cortical echotexture and loss of corticomedullary differentiation. These features were suggestive of CKD (Fig. 5). No other abnormalities were noticed at the first examination.

According to the clinical and paraclinical evidence, he met all the six cardinal or primary criteria and three minor or secondary criteria (Table 1) necessary for the diagnosis of BBS. On further inquiry, it was found that his younger sister also has similar symptoms. No genetic testing for BBS was available at our hospital.

**Table 1:** Diagnostic criteria for Bardet-Biedl syndrome

Primary features	Secondary features
Retinal dystrophy	Behavioral problems
Developmental delay	Neurological problems
Postaxial polydactyly	Hypogonadism
Truncal Obesity	Speech disorder
Renal abnormalities	Brachydactyly, syndactyly, or clinodactyly
Learning disabilities	Dental anomalies
	Nephrogenic diabetes insipidus
	Diabetes mellitus
	Hypertension
	Anosmia

Currently, the patient presented to the emergency department with chief complaints of fever, cough, vomiting, and decreased urine output for 7 days. The fever was associated with chills, the maximum temperature was undocumented. Vomiting was nonprojectile and vomitus consisted of ingested food particles. Cough was nonproductive initially, but was productive in the last day, with purulent sputum. Urine output was around 500 ml, without hematuria or frothiness. Patient also complained of decreased appetite. Chest examination revealed expiratory wheeze with bilateral basal crackles. Pulse rate was measured at 75 beats per minute and blood pressure was measured to be 100/70 mmHg.

Urine routine examination showed plenty of white blood cells, no red blood cells and 1+ proteinuria. Urine culture showed growth of *Enterococcus faecalis*. Serum level of various CKD parameters were Na^+^ 145, K^+^ 4.2, Ca^++^ 5.6 mg/dl, PO4^---^ 5.3 mg/dl, iPTH 1136 pg/ml, vitamin D 11.9 nmol/l, and albumin 39 gm/l. ANA, c-ANCA, p-ANCA, dsDNA titers were negative. Thyroid function tests revealed hypothyroidism. Twenty-four hour Holter did not show significant bradycardia episodes except for physiological sinus bradycardia.

The patient was diagnosed with CKD and severe metabolic acidosis with fluid overload on the background of BBS. Additionally, he had moderate anemia of chronic disease (hemoglobin level 8.6 gm/dl, low iron, high ferritin, and normal TIBC). Hemodialysis via a perm catheter was initiated after the diagnosis. During the hospital stay, he developed septic shock which was managed with inotropes and intravenous antibiotics. His condition was stable, and he was discharged after 2 weeks of hospital stay and was advised to do hemodialysis twice weekly via the perm catheter. The patient is currently undergoing hemodialysis twice a week at the same center and his parents have been counseled about the probable need of renal transplant. Additionally, the parents were counseled to give him low salt diet and avoid excess of fluids. The patient is currently under follow up with the nephrology department.

## Discussion

BBS is a rare autosomal recessive disease characterized by retinal dystrophy, obesity, postaxial polydactyly, renal defects, learning disabilities, and hypogonadism^1,6,7^. There are many more associated secondary BBS characteristics such as developmental delay, speech deficit, brachydactyly or syndactyly, dental defects, ataxia or poor coordination, olfactory deficit, diabetes mellitus, and congenital heart disease^7^. These associated minor features can be helpful in making a diagnosis and are important in the clinical management of BBS, which is limited to symptomatic treatment^1,6,7^. Most of these symptoms may not be present at birth but appear and progressively worsen during the first and second decades of life^7^.

The diagnosis is based on clinical findings and can be confirmed by sequencing known disease-causing genes in 80% of patients^1^. Genetic diagnosis of BBS is complicated due to the lack of gene-specific disease symptoms; however, it is gradually becoming more accessible with the invention of multigene sequencing technologies^7^. The use of such next-generation sequencing technologies has accelerated the identification of novel genes and causative disease mutations in known genes^6^. At least 20 BBS genes have already been identified, and all of them are involved in primary cilia functioning^7^. BBS genes encode proteins that localize to the cilia and basal body and are involved in cilia biogenesis and function. Mutations lead to defective cilia accounting in part for the pleiotropic effects observed in BBS^1^. Mutations in known BBS genes account for ~70–80% of cases, and tri-allelic inheritance has been suggested in about 5%^6^.

A study in France found that the frequency of occurrence of renal disease in BBS is 82%, with almost one-third of young adults presenting with signs compatible with CKD stages 2 and 3, which confirms that kidney involvement is one of the cardinal signs of the syndrome^8^. A study in Newfoundland, Canada found that 25% of patients had impaired GFR, and in the group with normal renal function, a further 20% had diffuse cortical loss with renal ultrasound. Impairment of GFR often occurs at an early age. By age 48 years, 25% of Bardet–Biedl patients had renal impairment. The earliest age of onset of chronic renal impairment was 2 years of age^9^. A study from United Kingdom suggests that the onset of primary renal disease in children predominantly occurs in infancy, and that patients with BBS primarily either develop CKD stages 4–5 in childhood or remain entirely or relatively free of severe renal disease. Around 8% of patients go on to develop end-stage renal disease requiring dialysis or transplantation^5^.

The exact pathogenesis behind the renal manifestation in BBS has not been identified. Ciliary dysfunction in BBS is believed to cause concentration defect in the nephrons. The water handling is impaired due to mistrafficking of aquaporin-2^10^. Because of impaired ciliary activity various intracellular homeostatic pathways such as noncanonical Wnt pathway is impaired in BBS leading to the development of cystic kidney disease^10,11^. Furthermore, impaired actin cytoskeleton regulation is believed to cause renal dysplasia in a patient of BBS^12^.

## Conclusion

BBS is currently treated symptomatically focusing on aggressive management of diabetes, hypertension, and metabolic syndrome to minimize the secondary impact to the kidney and eyes. Furthermore, genetic screening is required in families with BBS so that early preventive action can be taken.

## Ethical approval

Ethical approval not required.

## Consent

Written informed consent was obtained from the patient for publication of this case report and accompanying images. A copy of the written consent is available for review by the Editor-in-Chief of this journal on request.

## Sources of funding

None.

## Author contribution

P.P., U.P., and S.S.: conceptualization; U.P., S.S., P.P., and K.K.: writing – original draft; P.P., K.K., and M.S.: writing – review and editing; P.P.: visualization and supervision. All authors have read and agreed to the final version of the manuscript.

## Conflicts of interest disclosure

The authors declare no conflicts of interest.

## Research registration unique identifying number (UIN)

Research registration not required.

## Guarantor

Pashupati Pokharel.

## Provenance and peer review

Double anonymized.
